# Living with Cancer under watch and wait: a qualitative systematic review and thematic synthesis

**DOI:** 10.3389/fpsyg.2026.1830770

**Published:** 2026-10-05

**Authors:** Clement Boutry, Melissa Covington, Anika Kochar, Angel Nicholson, Naomi Thorpe, Nima Moghaddam

**Affiliations:** 1 Institute of Mental Health, School of Medicine, University of Nottingham, Nottingham, United Kingdom; 2 Nottinghamshire Healthcare NHS Foundation Trust, Nottingham, United Kingdom; 3 Psychological Health and Wellbeing Group, University of Lincoln, Lincoln, United Kingdom

**Keywords:** cancer treatment, psycho oncology, qualitative synthesis, thematic analysis, watching wait

## Abstract

**Objective:**

Watchful waiting, sometimes referred to as active surveillance or observation, is used across a range of cancer contexts to avoid or delay unnecessary treatment and its associated harms. Although this approach may reduce overtreatment, it can also place patients in the unusual position of living with a known cancer that is being monitored rather than immediately treated. This may have important psychological and social consequences. The aim of this review was to synthesize qualitative research exploring patients’ experiences of watchful waiting across cancer contexts.

**Methods:**

We conducted a qualitative systematic review and thematic synthesis of published studies examining patients’ experiences of watchful waiting. Systematic searches were undertaken across major electronic databases and supplemented by hand searching. Eligible studies used qualitative methods and focused on the experiences of adults living with cancer under a watchful waiting, active surveillance, or equivalent monitoring pathway. Included studies were appraised for methodological quality, and findings were analysed using thematic synthesis to generate descriptive and higher order analytical themes.

**Results:**

Twenty five studies were included, representing a range of cancer types and clinical pathways. Five overarching themes were identified. First, patients described complex emotional responses, including shock, fear, anxiety, uncertainty, and, for some, gradual adaptation over time. Second, decision making was shaped by perceptions of risk, beliefs about treatment, and varying degrees of trust in clinicians and healthcare systems. Third, social experiences were often marked by isolation, misunderstanding, stigma, and strain within family and intimate relationships, particularly where others struggled to understand distress in the absence of active treatment. Fourth, patients drew on a range of coping strategies, including reassurance from monitoring, peer support, information seeking, acceptance, and routines. Fifth, communication and supportive care emerged as central, with patients valuing clear explanations, continuity, validation, and opportunities to discuss uncertainty and emotional burden.

**Conclusion:**

Watchful waiting is not a passive experience. It can be psychologically demanding and socially complex, even when clinically appropriate. Cancer services should recognize the emotional burden associated with living with untreated but monitored disease and should proactively identify patients who may benefit from clearer communication, reassurance, and tailored psychological or peer support.

## Background

Over 3 million people live with cancer in the UK. A figure that is on the rise and expected to reach 4 million by 2030 with almost 400,000 new cancer diagnosed each year (Macmillan Cancer Support, 2025). These figures are particularly important considering the death associated with cancer, estimated at 167,000 yearly in the UK^1^. Besides, patients in cancer care experience a significantly lower quality of life compared to the general population, with heighted risk of depression, anxiety, and social dysfunction in addition to pain and reduced physical function (Marzorati et al., 2025; Bergerot et al., 2024). As a result, those patients in cancer care are at significant increased risk of suicide (Henson et al., 2019). Besides, such difficulties may have a direct negative impact on the effectiveness and cost of healthcare treatments (Bergerot et al., 2024; Mental Health Foundation, 2018; Ungvari et al., 2026) rendering them an important consideration.

Cancer manifests in diverse forms and severities, necessitating various treatment approaches. Chemotherapy and radiotherapy are among the most common and effective treatments (Baskar et al., 2012; Webster et al., 2025). However, these therapies are often associated with severe side effects, including cardiotoxicity, nephrotoxicity, myelosuppression, neurotoxicity, hepatotoxicity, gastrointestinal toxicity, mucositis, and alopecia. These side effects can significantly impact the quality of life and may lead to long-term post-cancer complications (Liu et al., 2021). Besides, surgery is also a form of treatment as a stand-alone one or in complement of others and may involve further risks and complications and impact quality of life, body image and mental health in general on a short and sometimes long term basis (Battistello et al., 2024; Collins et al., 2011).

To avoid these significant treatment-related burdens, especially for cancers where factors include incurability and indolence, some forms of the disease do not require immediate treatments (Blood Cancer UK, 2024). This approach is referred to as “Watch and Wait” (W&W), observation or active surveillance and entails regular check-ups with the clinical team at set intervals to monitor the disease and its progression with treatment offered if thought more effective (National Institute for Health and Care Excellence, 2016). While the precise definitions of these terms can vary by cancer type and protocol, this review uses W&W as an overarching term to encompass all non-interventional, monitoring-based management approaches. Th W&W approach provides the advantage of avoiding significant morbidity and mortality risks associated with some treatments while maintaining quality of life (Habr-Gama et al., 2015). However, patients often find this intervention perplexing, leading to concerns about not receiving immediate treatment (AlFayyad et al., 2020).

The W&W approach may worsen mental health for patients in cancer care. The psychological impact of cancer on patients is considerable with reported mood disorders and anxiety alongside social and the professional repercussions (Jarrett et al., 2013; Kanellopoulos et al., 2013; Timperi et al., 2013; Grassi et al., 2023). Those in cancer care are three times more likely to experience mental health difficulties (Linden et al., 2012) which are often unreported as considered as normal given the cancer circumstances (Naylor et al., 2016). What is more, a recent meta-synthesis identified that patients in the W&W pathways required more information and peer and emotional support (Charlick et al., 2019) as they are at greater risk of experiencing mental health difficulties when compared to the wider cancer population (Russell et al., 2022).

Existing evidence on the patient experience of W&W indicates an emotional burden associated with anxiety about tumor growth, recurrence, and the possibility of surgery (Pennings et al., 2021). Patient reports highlight unmet needs in understanding the W&W process and receiving reassurance, both of which are crucial for decision-making (Kim et al., 2018). Davies and colleagues (Davies et al., 2019) found that for thyroid cancer patients, concerns about W&W decrease over time and overall satisfaction with the approach is reported. However, qualitative studies on patient experiences often focus on specific types of cancer, limiting the generalizability of findings making generalization difficult.

There is a critical need to document the experiences of patients undergoing the W&W pathway across various types of cancer to fully understand their needs and determine the best ways to support them. Each type of cancer presents unique challenges, and the emotional and psychological impacts of W&W can vary significantly. By gathering comprehensive data on patient experiences, we can identify common themes and unique concerns that may not be apparent when focusing on a single type of cancer. Such comprehensive reports are essential as they can inform the development of guiding principles for psychological interventions tailored to support patients on W&W pathways. These interventions could address unmet needs, alleviate anxiety, and provide reassurance, ultimately improving the quality of life and mental health outcomes for these patients.

In this review, we aim to explore the overall patient experience of W&W across different cancer types. We will analyze and report possible common factors that influence patient experiences, such as emotional burdens, informational needs, and levels of satisfaction. By identifying these commonalities, we hope to contribute valuable insights that can shape supportive care strategies and enhance the decision-making process for both patients and healthcare providers.

## Methods

### Review design

This study was designed as a qualitative systematic review and thematic synthesis of published qualitative research exploring the experiences of adults living with cancer under watchful waiting, active surveillance, observation, or equivalent monitoring-based pathways. For consistency, the term W&W is used throughout this review as an overarching term to refer to these non-interventional, monitoring-based approaches. The review was registered with PROSPERO (Registration no. 529161).

### Search strategy

A systematic search was conducted to identify peer-reviewed qualitative studies exploring the experiences of patients with cancer undergoing a W&W approach. The search strategy was informed by the aim of the synthesis and developed by a Senior Information Specialist in collaboration with the research team. The following databases were searched: MEDLINE, APA PsycInfo via Ovid, and CINAHL Plus via EBSCOhost. The search strategy incorporated truncation, Boolean operators and database-specific subject headings to identify studies relating to cancer, W&W or equivalent monitoring approaches, psychological or experiential outcomes, and qualitative methods. Full search terms for each database are provided in Appendix 1.

### Eligibility criteria

Studies were eligible for inclusion if they:

1. Included adult participants aged 18 years or older;
2. Focused on participants with a formal diagnosis of any type of cancer;
3. Examined experiences of W&W, active surveillance, observation, active monitoring, or an equivalent monitoring-based pathway;
4. Presented original primary qualitative data; and
5. Explored patients’ psychological, emotional, social, or experiential responses to Living under a W&W approach.

Studies were excluded if they did not focus on adults with cancer, did not include a W&W or equivalent monitoring-based pathway, did not report primary qualitative findings, or did not present patient experiences in a way that could be distinguished from other participant groups. Studies were also excluded if they were not published in English. Where studies included clinicians, partners, caregivers or mixed participant groups, they were retained only if findings relating to patients living under W&W were clearly reported or distinguishable.

### Study selection

Search results were de-duplicated and exported into Rayyan, a web-based platform designed to support the organization and screening of records in systematic reviews. Titles and abstracts were screened against the eligibility criteria, followed by full-text review of potentially relevant articles. No artificial intelligence-assisted screening functions within Rayyan were used; eligibility decisions were made by members of the research team. Disagreements or uncertainties about eligibility were resolved through discussion within the research team. The study selection process is reported in the PRISMA diagram (Figure 1).

**Figure 1:**
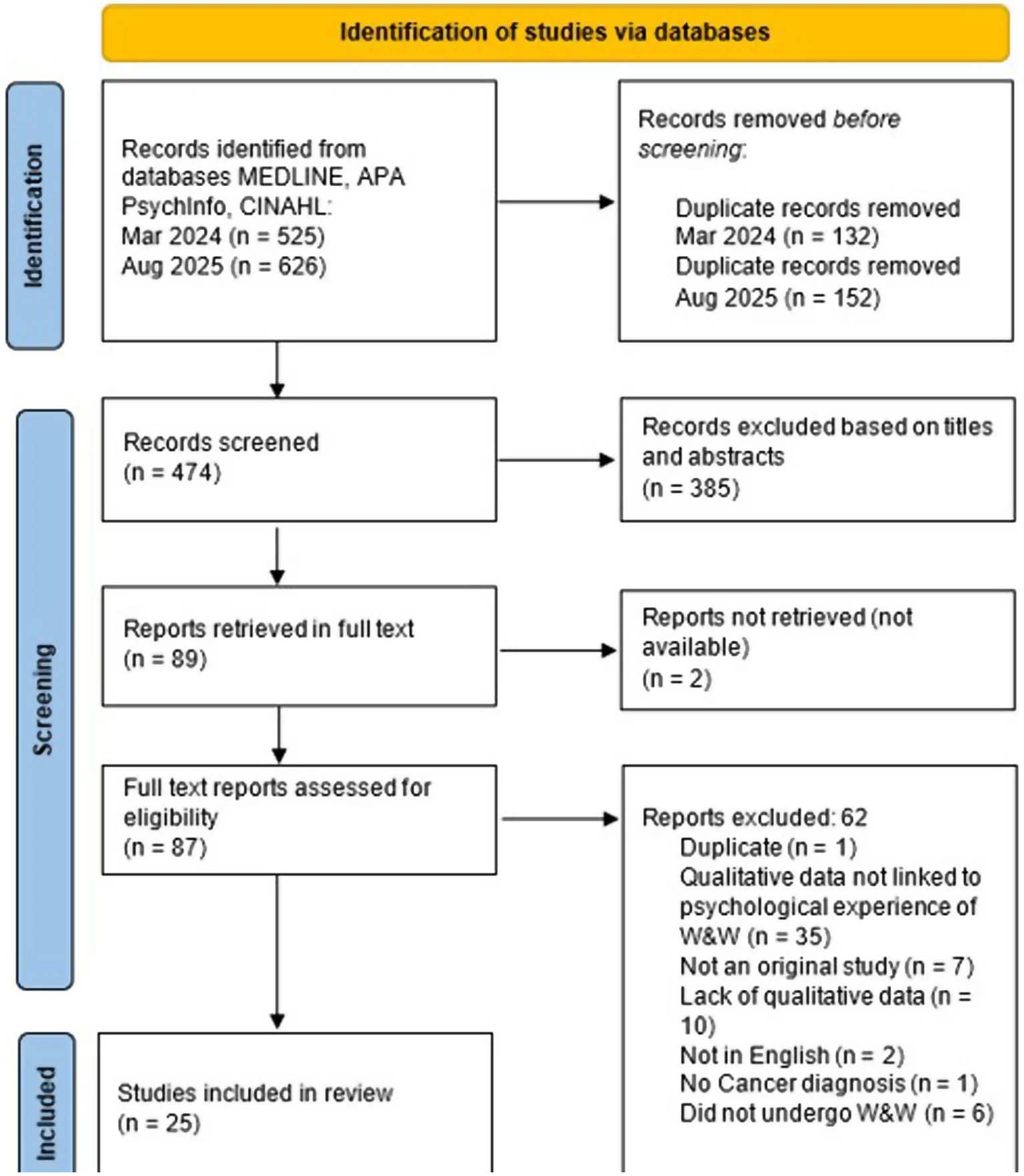
PRISMA diagram.

### Quality appraisal

All included studies were assessed for methodological quality using the Critical Appraisal Skills Programme (CASP) (Critical Appraisal Skills Programme, 2018) qualitative checklist. The appraisal was undertaken to describe the quality and reporting of the included studies rather than to exclude studies or weight their contribution to the synthesis. As the included studies were of broadly similar quality, all studies were integrated equally within the analysis. The full quality appraisal is provided in Appendix 2.

### Data extraction

Data were extracted using the Inclusive Extraction of Qualitative Findings approach. This involved extracting the full findings from each included study, including both participant accounts and authors’ interpretations. This approach was selected to preserve the richness and interpretive depth of the primary qualitative studies and to enable synthesis across different cancer types and W&W contexts.

### Data analysis and synthesis

Ensuring a comprehensive and nuanced synthesis, the Inclusive Extraction of Qualitative Findings method (Noyes et al., 2018) was applied to extract data. It involves incorporating the full findings from each included study, including both participant accounts and the authors’ interpretations. This ensured a comprehensive and nuanced synthesis.

Subsequently, the extracted findings were uploaded into NVivo, qualitative data-management software used to organize, code and retrieve textual data, and were analysed using the thematic synthesis methodology outlined by Thomas and Harden (2008). No artificial intelligence-assisted functions within NVivo were used in the analysis. Coding, organization of codes, and the development of descriptive and analytical themes were undertaken by the research team. An inductive process was adopted, beginning with line-by-line coding of extracted text in NVivo to capture meaning and content. Codes were then iteratively organized into descriptive themes by translating concepts across studies, identifying similarities and differences, and constructing a hierarchical framework. Finally, we developed analytical themes that moved beyond the content of the primary studies, using the descriptive themes as a foundation to generate higher-order insights. The synthesis therefore integrates patient narratives with interpretive layers from the original authors, while also providing new interpretations that extend beyond the primary material.

## Results

A total of 474 unique studies were identified. Following title and abstract screening, 84 articles were retained for full-text review. Of these, 25 met the eligibility criteria and were included in the synthesis (see Figure 1).

I ncluded studies were published between 2007 and 2025; this range reflects the publication dates of studies that met the eligibility criteria, rather than a predefined date restriction (see Table 1 for study characteristics). Of these, nine (36.0%) were conducted in the UK and seven (28.0%) in the US, with the remainder originating from The Netherlands, Canda, Greece, Australia and Ireland. Data collection methods included interviews (92.0%), focus groups (8.0%). While nearly all studies focused exclusively on the target population, several also included patients’ partners (*n* = 1) and clinicians (*n* = 1); however, these were retained as findings on the target population were clearly reported or distinguishable.

**Table 1:** Selected articles and characteristics.

Authors	Title	Year	Country of origin	Demographics	Cancer type	Method	Analysis
Bashir Al Hussein Al Awamlh, Christopher J. D. Wallis, Carolyn Diehl, Daniel A. Barocas, Laura M. Beskow	The lived experience of prostate cancer: 10-year survivor perspectives following contemporary treatment of localized prostate cancer	2023	USA	66 males; 12 < 55, 54 > 55; 50 White/Caucasian, 9 Black/African-American, 1 Latino/Hispanic/Mexican-American, 1 Asian/Oriental/Pacific Islander, 1 American Indian/Native Alaskan, 2 Other, 2 Unavailable; Approximately one-fifth diagnosed before age 55.	Prostate	Semi-structured interviews	Grounded theory approach
Donald E Bailey Jr., Meredith Wallce, Merle H Mishel	Watching, waiting and uncertainty in prostate cancer	2007	USA	10 males aged between 64–88; 2 African-American, 8 Caucasian; Time since diagnosis ranged from 4–12 months.	Prostate	Semi-structured interviews	Content analysis
Kerri Beckmann, Declan Cahill, Christian Brown, Mieke Van Hemelrijck, Netty Kinsella	Understanding reasons for non-adherence to active surveillance for low-intermediate risk prostate cancer	2021	UK	14 males aged between 49 and 76 years, average 64 years; 10 White European, 1 Asian, 1 Black African, 1 Black Caribbean; 7 married, 3 divorced, 2 widower, 1 single, 1 partnership; Months on AS 25 to 63 months, average 39 months.	Prostate	Semi-structured interviews	Inductive thematic analysis
Zackary D. Berger, Jonathan C. Yeh, H. Ballentine Carter, Craig Evan Pollack	Characteristics and Experiences of Patients with Localized Prostate Cancer Who Left an Active Surveillance Program	2014	USA	21 males. 14 elected to leave AS, 7 left following physician’s recommendation. Characteristic data not provided due to small sample size.	Prostate	Semi-structured interviews	Thematic analysis
Geneviève Charbonneau, Catherine Hudson, Luke T. Lavallée, Wassim Kassouf, Isabelle Gaboury, Paul Toren, Félix Couture, Véronique Foley, Amélie Tétu, Patrick O. Richard	Patients’ perceptions on active surveillance for the treatment of small renal masses	2022	Canada	24 interviewees, 16 treated with definitive treatment, 5 currently on AS, 3 caregivers; 13 males, 11 females aged 41–78.	Renal	Focus group discussions using semi-structured interview guide	Thematic analysis
Raymond G. Deobald, Eva S. W. Cheng, Yoo-Joung Ko, Frances C. Wright, Paul J. Karanicolas	A qualitative study of patient and clinician attitudes regarding surveillance after a resection of pancreatic and peri-ampullary cancer	2015	Canada	15 individiuals, 8 males, 7 females with a diagnosis of cancer and 7 clinicians; Age range 43–84; 13 Caucasian, 1 Korean, 1 West Indian; Demographics not provided for clinicians.	Pancreatic	Semi-structured interviews	Iterative approach
Kim Donachie, Erik Cornel, Marian Adriaansen, Rosa Mennes, Inge van Oort, Esther Bakker, Lilian Lechner	Optimizing psychosocial support in prostate cancer patients during active surveillance	2020	Netherlands	17 males; Age range 54–76, average 67; All Caucasian and Dutch descent; All heterosexual; 16 married; Spouse present for 11 interviews; Average time since diagnosis 47 months (<1 month - 120 months); All had post-high school education with majority (58%) having a master’s or bachelor’s degree.	Prostate	Semi-structured interviews	Sequential
Claire Dunnion, Martina Giltenane, Maura Dowling	The ‘inbetweeners’: living on a watch and wait approach for chronic lymphocytic leukaemia – a qualitative study	2023	Ireland	7 interviewees, 6 male, 1 female; All aged 50+; 5 on W&W for 4–6 years, 2 on W&W for 8–10 years; All married; 4 in full-time employment, 1 early retirement.	Lymphocytic Leukaemia	Interviews	Systematic text condensation
J. Evans, S. Ziebland, A. R. Pettitt	Incurable, invisible, and inconclusive: watchful waiting for chronic lymphocytic leukaemia and implications for doctor-patient communication	2011	UK	12 interviewees, 4 male, 8 female; Age range 54–87; Time on W&W ranged from 20 months to 7 years; 11 married, 1 widowed.	Lymphocytic Leukaemia	Narrative and semi-structured interviews	Thematic analysis
Omar Eymech, Oliver Brunckhorst, Louis Fox, Anam Jawald, Mieke Van Kemelrijck, Robert Stewart, Prokar Dasgupta, Kamran Ahmed	An exploration of wellbeing in men diagnosed with prostate cancer undergoing active surveillance: a qualitative study	2022	UK	13 males; Age range 57–74, average 66 years; Time since diagnosis between 1 and 7 years, average 4 years; 10 married, 3 single; 7 retired, 6 employed; All heterosexual; 6 Caucasian, 4 Black Carribean, 2 Black African, 1 Mixed White-Vietnamese.	Prostate	Semi-structured interviews	Inductive thematic analysis
Merel Hermus, Berend J. van der Wilk, Rebecca T. H. Chang, Gerlise Collee, Bo J. Noordman, Peter-Paul L. O. Coene, Jan Willem T. Dekker, Henk H. Hartgrink, Joos Heisterkamp, Grard A. P. Nieuwenhuijzen Camiel Rosman, Liesbeth Timmermans, Bas P. L. Wijnhoven, Charlène J. van der Zijden, Jan J. Busschbach, J. Jan B. van Lanschot, Sjoerd M. Lagarde, Leonieke W. Kranenburg	Patient preferences for active surveillance vs. standard surgery after neoadjuvant chemoradiotherapy in oesophageal cancer treatment The NOSANO-study	2022	Netherlands	40 interviewees, 12 male, 15 female; 20 under active surveillance group, 20 standard surgery group; Age range for AS group 55–78, age range for surgery group 51–79.	Oesophageal	Interviews	Grounded theory approach
Dimitrios Kyrou, Konstantina Stavrogianni, George Koulierakis, Nikolaos Vrontaras, Kostas Stamatopoulos, Christina Karamanidou	The Looming Cancer: A Qualitative Study on the Experience of Living with Chronic Lymphocytic Leukemia (CLL) before the Initiation of Treatment	2024	Greece	8 interviewees, 4 male, 4 female; Age range 60–75, one 40 years old; Time since diagnosis ranged from 6 months to 9 years.	Lymphocytic Leukaemia	Semi-structured interviews	Interpretative phenomenological analysis (IPA)
Devon M. Langston, Matthew J. DePuccio, Ann Scheck McAlearney, Sooyoung Kim, Alice A. Gaughan, Alicia Scimeca, Shawn Dason, Tasha Posid, and John O. DeLancey	Assessment of Patient Experience During Active Surveillance for Prostate Cancer	2025	USA	27 interviewees aged between 51 and 83 years, average 69 years; 14 male and 13 female; 26 white and 1 black; time since diagnosis ranged from 62 days to over 7 years.	Prostate	Semi-structured interviews	Thematic analysis
Emily M. Mader, Hsin H. Li, Kathleen D. Lyons, Christopher P. Morley, Margaret K. Formica, Scott D. Perrapato, Brian H. Irwin, John D. Seigne, Elias S. Hyams, Terry Mosher, Mark T. Hegel, Telisa M. Stewart	Qualitative insights into how men with low-risk prostate cancer choosing active surveillance negotiate stress and uncertainty	2017	USA	15 males; Average age 65; All White Non-Hispanic; 11 married, 1 divorced, 3 never married; 7 full time employed, 7 retired, 7 declined to answer.	Prostate	Semi-structured interviews	Thematic analysis
Lauren Matheson, Sarah Wilding, Richard Wagland, Johana Nayoan, Carol Rivas, Amy Downing, Penny Wright, Jo Brett, Therese Kearney, William Cross, Adam Glaser, Anna Gavin, Eila Watson	The psychological impact of being on a monitoring pathway for localized prostate cancer: A UK-wide mixed methods study	2019	UK	24 males; Age range <55–85+; 22 heterosexual, 2 gay; 22 White, 2 Black; 18 married, 2 cohabiting, 1 divorced, 2 widowed, 1 single; Time since diagnosis ranged from 18 to 41 months.	Prostate	Semi-structured interviews	Thematic analysis
Dorothy McCaughan, Eve Roman, Rebecca Sheridan, Ann Hewison, Alexandra G. Smith, Russell Patmore, Debra A. Howell	Patient perspectives of ‘Watch and Wait’ for chronic hematological cancers: Findings from a qualitative study	2023	UK	35 interviewees, 19 male, 16 female; Age range 54–86; 7 began and stayed on W&W, remainder received treatment at least once.	Hematological	Semi-structured interviews	Thematic content analysis
Megan McIntosh, Melissa J. Opozda, Michael O’Callaghan, Andrew D. Vincent, Daniel A. Galvão, Camille E. Short	Why do men with prostate cancer discontinue active surveillance for definitive treatment? A mixed methods investigation	2022	Australia	33 males; Age range 48–75; 10 still on AS, 23 left and undergone definitive treatment; Most were married (82%), retired (61%), educated beyond high school (79%) and lived in a major city (64%); Average time on W&W 3.3 years.	Prostate	Semi-structured interviews	Reflexive thematic analysis
Alexander J. Pennings, Merel L. Kimman, Anke H. C. Gielen, Geerard L. Beets, Jarno Melenhorst, Stephanie O. Breukink	Burden of disease experienced by patients following a watch-and-wait policy for locally advanced rectal cancer: A qualitative study	2021	Netherlands	18 interviewees; 14 male, 4 female; Age range 52–83, average 68 years; Time on W&W 5–71 months, average 35 months.	Rectal	Semi-structured interviews	Thematic analysis
Ruth Reeve, Claire Foster, Lucy Brindle	Exploring patient experiences of surveillance for pancreatic cystic neoplasms: a qualitative study	2024	UK	12 males aged between 57 and 74 years, median 69 years; predominantly white, retired and married; Median time since diagnosis 3 years.	Pancreatic	Semi-structured interviews	Reflexive thematic analysis
Katie Russell, Anna Tickle, Nima Moghaddam, Sanchia Biswas	Exploring the Psychosocial Needs of Adults with Hematological Cancer under Watch-and-Wait: A Qualitative Study	2023	UK	15 interviewees at T1, 12 females, 3 males; 12 at T2, 9 females, 3 males; Age range 51–71, average 62 years; 14 White British, 1 Mixed Heritage; 2 employed, 3 self-employed, 8 retired, 2 unemployed; Time on W&W 0.25–11 years.	Hematological	Semi-structured interviews	Inductive thematic analysis
Aaron T. Seaman, Kathryn L. Taylor, Kimberly Davis, Kenneth G. Nepple, John H. Lynch, Anthony D. Oberle, Ingrid J. Hall, Robert J. Volk, Heather Schacht Reisinger, Richard M. Hoffman	Why men with a low-risk prostate cancer select and stay on active surveillance: a qualitative study	2019	USA	21 males; Age range 56–84, average 70.4 years; 18 White, 2 Black, 1 Other Ethnicity; 16 in Active Surveillance, 5 in Active Treatment.	Prostate	Semi-structured interviews	Thematic analysis
Jason D. Tan, Phyllis N. Butow, Frances M. Boyle, Robyn P. M. Saw, Amanda J. O’Reilly	A qualitative assessment of psychosocial impact, coping and adjustment in high-risk melanoma patients and caregivers	2014	Australia	19 interviewees, 9 male, 10 female; Age range 30–82, average 58 years; Years since diagnosis 0.6–3.1, average 1.7; 14 caregivers interviewed as well, 3 male, 11 female; Age range 31–67, average 57 years; 8 partners of patient, 1 parent, 1 child, 2 friends, 2 neither friends nor family.	Melanoma	Semi-structured interviews	Thematic analysis
Julia Wade, Jenny Donovan, Athene Lane, Michael Davis, Eleanor Walsh, David Neal, Emma Turner, Richard Martin, Chris Metcalfe, Tim Peters, Freddie Hamdy, Roger Kockelbergh, James Catto, Alan Paul, Peter Holding, Derek Rosario, Howard Kynaston, Edward Rowe, Owen Hughes, Prasad Bollina, David Gillatt, Alan Doherty, Vincent J Gnanapragasam, Edgar Paez	Strategies adopted by men to deal with uncertainty and anxiety when following an active surveillance/monitoring protocol for localized prostate cancer and implications for care: a longitudinal qualitative study embedded within the ProtecT trial	2020	UK	20 males; Age range 52–68; 12 randomly allocated to Active Monitoring, 8 chose Active Monitoring.	Prostate	Interviews	Thematic analysis
Renda Soylemez Wiener, Michael K. Gould, Steven Woloshin, Lisa M. Schwartz, Jack A. Clark	The thing is not knowing’: patients’ perspectives on surveillance of an indeterminate pulmonary nodule	2012	USA	22 interviewees, 3 male, 19 female; Average age 60.7 years; 77% White, 18% Black, 4.5% Hispanic; Time since diagnosis 2–28 months.	Pulmonary	Focus groups	Inductive grounded theory
Anna Zanotto, Karen Goodall, Marion Ellison, Chris McVittie	‘Make Them Wonder How You Are Still Smiling’: The Lived Experience of Coping With a Brain Tumor	2023	UK	12 interviewees, 2 males, 10 females; Age range 29–54, average 42 years; Time since diagnosis 15 months to 8 years 1 month, average 3.5 years.	Brain	Semi-structured interviews	Interpretative phenomenological approach (IPA)

Five overarching themes that capture the lived experience of patients undergoing W&W were identified (Table 2). First, *emotional and psychological responses* highlighted the fluctuating states of fear, anxiety, acceptance, and identity struggles that shaped patients’ adjustment over time. Second, *understanding and decision-making around W&W* revealed how choices were influenced by risk perceptions, trust in clinicians, and comparisons with active treatment. Third, *relationships and social experience* encompassed feelings of misunderstanding, concealment, and the critical role of family, peers, and healthcare professionals in providing support. Fourth, *living with uncertainty* reflected ongoing tensions between reassurance and loss of control, particularly in relation to monitoring schedules and limited information. Finally, *coping, control, and life adjustments* illustrated how individuals redefined priorities, engaged in lifestyle changes, and reframed cancer as a manageable condition in order to restore a sense of agency and quality of life. Together, these themes illustrate the complex and evolving psychosocial landscape of W&W, underscoring the need for holistic support that goes beyond clinical monitoring.

**Table 2:** Themes and subthemes.

Emotional and psychological response to WW	Understanding and decision-making around WW	Relationships and social experience	Living with uncertainty	Coping, control, and life adjustments
Initial shock and disbelief	Choosing WW over active treatment	Feeling misunderstood or isolated	Lack of control, oscillation between calm and fear	Lifestyle adjustments and health behaviors
Ongoing anxiety and fear of progression or recurrence	Influence of clinicians, family, peers, and second opinions	Minimisation by others	Living between illness and wellness	Reframing cancer and cultivating acceptance
Identity struggles and imposter feelings	Counterintuitive perceptions of WW	Non-disclosure and social concealment	Acute anxiety from monitoring procedures and results	Seeking agency through information or planning
Acceptance and emotional adaptation over time	Regret, re-evaluation, or switching to active treatment	Peer and family support	Psychological cycle of surveillance	Reprioritising life and identity
Coping through denial and avoidance				

Illustrating quotes for each theme/subtheme can be found in Table 3.

**Table 3:** Illustrative quotes.

Theme	Sub-theme	Quote
Emotional and psychological response to WW	Initial shock and disbelief	“While the diagnosis of PCa was sometimes described as an emotionally difficult period involving ‘shock’ and ‘worry’, most men appeared to have adjusted positively to both their diagnosis and being on AS/WW” (FO)^1^ “I know I do not have anything. It must be a mistake, just a shadow. But it’s there, it’s there. You have to deal with it. … It’s a painful diagnosis” (FO)^2^ “We did expect treatment to be given straight away. I was scared stiff that that would be it, I would lose my hair and I’d be dead within 5 years” (FO)^3^
	Ongoing anxiety and fear of progression or recurrence	“It’s nerve-wracking just to sit and wait, … wondering when the bomb is going to drop” (FO)^2^ “You’ve got this thing growing inside you… I feel my life has been invaded, I view the cancer as like, an invasion of my life”(FO)^4^ “The fearful anticipation of tumor growing, or symptoms worsening, was described as a feeling of ‘having this ticking time bomb, just waiting’ (May)” (SO)^5^ “It does not matter how far away you get from diagnosis stage. you never really feel like you are in the clear” (FO)^6^
	Identity struggles and imposter feelings	“We are kind of stuck together for the rest of our lives… yes. there is something in me now that is not going anywhere” (FO)^5^ “Individuals appeared to rationalize the experiences of having the cancer minimized as being a result of ‘not looking like a cancer patient’ and not having anticancer treatments” (SO)^7^ “I’ve got as much information as I can possibly gather. … And I’m not ill, but I am ill. … all the friends that I know that have got cancer, they are ill, and we keep losing them … And I’ve got it, but it does not show, it does not affect me. … Well, how can you accept it? You cannot just accept it” (FO)^3^
	Acceptance and emotional adaptation over time	“I’ve grown to accept it. As time’s gone on, you just sort of accept it more… put it to the back of your mind as much as you can” (FO)^1^ “Some participants appeared to have passed through various stages of mourning and reached disease acceptance… Although uncertainty and anxiety was still experienced occasionally, the emotional discomfort … was openly discussed” (FO)^8^ “You kind of become more accepting that you are going to die. … dealing with that just relaxed me a bit” (FO)^5^ “As time goes by and nothing is getting worse I’m generally more reassured and I just carry on with life as per normal” (FO)^1^
	Coping through denial and avoidance	“I try to forget about this, because if I put it in my head, I’m going to be like her, crying all day and depressed” (FO)^2^ “I never think: ‘oh my goodness, I’ve got prostate cancer’ other than talking with you [or] when I fill in medical questionnaires” (FO)^1^ “Some men opted to minimize or redefine the cancer threat… believing that the cancer might remain unchanged for a long period of time, … and they were likely to die from a heart attack or stroke before the cancer killed them” (FO)^9^
Understanding and Decision-making Around WW	Choosing WW over active treatment	“I get to keep my prostate. I do not know what it would be like if I did not have it. I know it’s an organ that’s used in going to the bathroom, and it’s also used in the other, the sexual part.” (FO)^10^ “It is major surgery, which comes with consequences about how life will be afterwards. That is my biggest fear; my quality of life has always been so good […] that cannot become better after surgery, it will only become worse. And that made me think: I’m not going to have this surgery.” (FO)^11^ “The knowledge of them feeling good in that moment, and the knowledge that surgery could negatively change their lives’ quality—in their opinion—made them confident in their decision to opt for active surveillance. They felt like it was a ‘responsible risk’ to refrain from surgery.” (SO)^11^ “Surgery is often referred to as the ‘final back-up option’ and considered as something they could undergo any time in the future anyway. (SO)^11^
	Influence of clinicians, family, peers, and second opinions	“I know they are busy people, but a lot of times when I go for a doctor visit, they have got so many people scheduled, they do not take enough personal time with their patient to talk freely and ask the right questions and voice concerns.” (FO)^10^ “You know, you are talking to a person, not a patient. You’re talking to somebody who has a problem. They come to you, they got a problem, and they are looking for some help.” (FO)^10^ “With my doctor we have the best relationship. I have had just two appointments as I am still in the beginning, but she is exceptional. Exceptional. And as time goes by, I want our relationship to become closer, warmer, not the typical relationship between doctor and patient.” (Jim, less than 1 year since diagnosis) (FO)^12^ “[Providers should] advise their patients to consult with different specialists before any decision was made, that would be better rather than appearing to know everything.” (FO)^10^
	Counterintuitive perceptions of WW	“There seems to be nothing going on, except from the white blood cell count. There is an underlying condition, but since the count is not high you are just fine. … Ahh, I felt calm, I ate, I drunk water (chuckles).” (Barbara, less than 1 year since diagnosis, FO)^12^ “They told me, ‘It is nothing, your health will be monitored and there is no need to be afraid,’ but such was the magnitude of my reassurance that I did not even realize CLL was a malignancy.” (George, 5 years since diagnosis, FO)^12^ “Their hematologist’s reassurance regarding the good prognosis of the disease was the greatest source of relief for participants, which consequently helped them overcome the initial shock and come to terms with their diagnosis.” (SO)^12^
	Regret, re-evaluation, or switching to active treatment	“Thinking about it all the time, that’s it. Thinking about it all the time. Did I make the right decision? Should I have went on ahead and got it done, get it over with?” (FO)^10^ “It turned out to be much more of a speed bump than a brick wall… I had a relative who was diagnosed exactly the same time as me and actually opted to go through the surgery and six months later, he was still just miserable and I was whole and feeling good.”“Some patients reported a fear of not waking up from anaesthesia, or they were afraid of complications.” (SO)^10^
Relationships and social experience	Feeling misunderstood or isolated	“This concealment, however, came at the expense of their own wellbeing. Men described being left with little social support, fighting their own battles without family or friend support, meaning an increased feeling of loneliness was felt.” (SO)^13^ “Other prostate cancer patients in the form of friends or even support groups were also a useful source of support as they felt ‘better understood’ (participant 13, Black African, age 67) and that they could ‘compare notes and experiences’ (participant 4, White, age 71).” (FO/SO)^13^ “Notably, Maria’s (3 years since diagnosis) family blocked the expression of negative emotions, which led her to shut herself off and go through them on her own.” (SO)^12^
	Minimisation by others	“Seeing peers in a worse condition and receiving therapy was another triggering condition, which made participants uncertain with regard to the way they would manage to cope if they ever ended up in their peers’ shoes.” (SO)^12^ “Media portrayals of cancer as dramatic and life-threatening further contributed to the perception that WW cancers were not ‘real cancers’.” (SO)^14^ “Some described peers or family saying they were ‘lucky,’ which, while well-meaning, made them feel dismissed and invisible.” (FO)^7^
	Non-disclosure and social concealment	“I know they would be very worried and panic. Unless it moves into a worse position, then I do not really want to have that family discussion just yet.” (participant 9, White/Vietnamese, age 60, FO)^13^ “I’m trying not to lay my problems on other people. And if I do not have problems, then hopefully they are not harboring them.” (participant 5, White, age 68, FO)^13^ “When I get emotional, I try not to let things really affect me (emotionally)… I do not want to make others pity me… maybe they might even avoid you.” (Maria, 3 years since diagnosis, FO)^12^
	Peer and family support	“They all have not got a supportive wife; without her, it would not have been such an experience for me where I felt confident about going forward. I think men who are on their own will find it much more difficult.” (participant 1, White, age 66, FO)^13^ “For many, the support of their partner was crucial throughout the process, with men describing how they would not be able to cope without them.” (SO)^13^ “Worry and attention from family members kept reminding her that she has CLL.” (SO)^12^
Living with uncertainty	Lack of control, oscillation between calm and fear	“Interestingly, the cancer diagnosis led to many becoming more aware of their body and the issues that impacted their physical wellbeing.”“Following the recognition of the impact of the disease, a somewhat normal state of mind returned to many.”“They preferred to leave the period of cancer behind and get on with their lives.” (SO)^13^
	Living between illness and wellness	“These physical problems frequently impacted other aspects of their lives and their social and mental wellbeing. This included affecting their relationships with their partner due to sexual dysfunction, reduced attendance at leisure activities because of fatigue and reduced social interactions secondary to the fear of embarrassment from an episode of urinary incontinence.” (SO)^13^ “Other participants believed these symptoms could also be due to cancer but are being ‘overshadowed’ by old age and other comorbidities.” (FO)^13^ “I missed a few days initially when I had the treatment, but other than that it does not affect my ability to do my job.” (FO)^10^
	Acute anxiety from monitoring procedures and results	“The worst bit was the actual waiting for the results. This plays tricks on your mind.” (participant 12, White, age 59, FO)^13^ “I did not look forward to the biopsies, which was once a year. I had several days of discomfort from that… It’s not something I was really excited about, but I just saw that as my best option at the time.” (FO)^10^ “I do not worry about it on a day-to-day basis or month-to-month basis. Every 6 months when I go to my check-up appointment, I get worried about it, but I get informed of what’s happening. And then we move on.” (participant 5, White, age 68, FO)^13^
	Psychological cycle of surveillance	“Several described learning to manage to continue with their daily lives without worrying about their disease. During this time, they did not speak about or address their prostate cancer.” (SO)^13^ “This was however temporary, with a repeated reminder about their illness through an upcoming PSA test or follow-up appointment.” (SO)^13^ “Participants lived in a constant cycle of a relapsing–remitting anxiety related to the increased obsession with their PSA value around the time of appointments.” (SO)^13^
Coping, control, and life adjustments	Lifestyle adjustments and health behaviors	“The things that I previously loved to eat, my wife cut it out. I began to start eating more green and leafy vegetables. The alcohol consumption went down… I get a lot of exercise and I stay mentally fit, as well as physically fit.” (FO)^10^ “Men started to seek online information about risk factors and lifestyle modifications… consuming less red meat or reducing alcohol intake.” (FO)^13^ “Several men increased their physical activity levels by going to the gym more, pursuing a new sport or simply by going on more walks.” (SO)^13^
	Reframing cancer and cultivating acceptance	“Me personally, I associated with, you know, imminent death. But … I realised that if I’m monitored properly, then I can live as long as I would live anyway.” (participant 11, Black Caribbean, age 60)“Many described the prospect of a low-grade disease was not as bad as initially believed, but still there was a recognition of what the diagnosis could potentially mean for their future lives.” (FO)^13^ “By choosing to postpone and hopefully avoid surgery, they have the feeling of control over the situation; at least they can ‘choose’ to maintain their quality of life.” (FO)^11^
	Seeking agency through information or planning	“Interviewees emphasized the importance of providers giving newly diagnosed patients complete information about their options… to put all the facts out there so that people know from the get go, what they are getting into.” (SO)^10^ “When I was first diagnosed, Dr. [Name] gave me a book on prostate cancer. … Most people do not take time to even read what the doctor gives them.” (FO)^9^ “Many became obsessed with their PSA levels, keeping meticulous records of their results… This gave some a sense of control … however, elevated results could quickly trigger an emotional disequilibrium again.” (SO)^13^
	Reprioritising life and identity	“Sometimes I think to myself hey, I’m a cancer overcomer. Let us just take a day at a time and enjoy life and every day is a gift.” (FO)^10^ “Some described their experience with prostate cancer as giving them ‘a greater appreciation for life’ … and a desire to ‘try to live a better life’ … discarding ‘the petty sort of worries that plague you as you go through life.” (FO)^10^ “I feel very good right now, and I want to enjoy my life now and seize the day.” (FO)^11^

FO, First Order (direct participant quote); SO, Second Order.

Some concepts, such as trust in clinicians, appeared across more than one theme; where this occurred, themes were distinguished according to the analytic role of the concept, for example decision-making in Theme 2 versus ongoing uncertainty management in Theme 4.

### Theme 1: emotional and psychological response to W&W

Patients described a wide spectrum of emotional and psychological responses to W&W. Some experienced profound fear and shock following diagnosis, while others reported ongoing anxiety about progression, struggles with identity, or attempts to cultivate acceptance. The absence of symptoms or active treatment often heightened these feelings, leaving patients caught between uncertainty and adaptation. This dynamic process is explored through five subthemes: initial shock and disbelief, ongoing anxiety and fear of progression or recurrence, identity struggles and imposter feelings, acceptance and emotional adaptation over time, and coping through denial and avoidance.

### Initial shock and disbelief

Diagnosis was often experienced as a profound rupture, marked by shock, fear, and disbelief. The absence of symptoms intensified this sense of unreality, as patients struggled to reconcile being otherwise healthy with the sudden label of “cancer.” The word itself carried heavy associations with death and suffering, leaving some “frozen in terror” despite reassurances of low risk. Others described feeling unprepared, overwhelmed and isolated, picturing the experience to a “rollercoaster” of emotions. This disruptive early phase often shaped how patients approached W&W in its early stages.

### Ongoing anxiety and fear of progression or recurrence

Beyond the initial shock, many reported a persistent background worry about cancer “growing inside them.” Everyday aches and pains were often interpreted as signs of progression, while the cyclical nature of monitoring amplified fears; waiting for results brought acute anxiety, and reassurance was often temporary. For some, living with unpredictability felt like carrying a “ticking time bomb.” Although anxiety ebbed and flowed, it rarely disappeared, influencing daily quality of life and highlighting the need for ongoing psychological support.

### Identity struggles and imposter feelings

The invisibility of illness created tension in how patients understood and communicated their identity of someone with cancer. Many felt like “frauds” or “drama queens” because they lacked symptoms or active treatment, making it harder to access social support. This “imposter” experience sometimes resulted in concealing their diagnosis, while those who disclosed it often felt misunderstood or dismissed. Living with cancer, even silently, disrupted self-perceptions, as patients struggled to reconcile the idea of being unwell with their outwardly normal lives. These identity tensions underscored the unique psychosocial burden of W&W, where cancer is both minimized and inescapable.

### Acceptance and emotional adaptation over time

For some, repeated experiences of stability fostered a gradual shift toward acceptance. This process unfolded unevenly but was supported by reassurance from clinicians, reframing W&W as a manageable alternative to active treatment, and personal coping strategies such as focusing on daily life. Patients spoke of learning to “live alongside” cancer, incorporating it into their sense of self without letting it dominate their identity. For some, this acceptance was supported by trust in healthcare professionals, while others emphasized personal coping strategies such as focusing on daily life or drawing meaning from their experiences. Acceptance, however, did not mean full resolution; moments of anxiety and vulnerability continued to resurface.

### Coping through denial and avoidance

A final pattern involved deliberate minimisation or avoidance of illness. Patients downplayed the seriousness of their diagnosis or avoided thinking about cancer to protect their sense of normality. While these strategies provided short-term relief, they sometimes created tension when monitoring or medical discussions forced patients to confront the reality of disease. Denial and avoidance thus functioned as double-edged tools as helping patients cope in the moment but limiting longer-term adjustment.

### Theme 2: understanding and decision-making around W&W

Patients’ decisions about W&W reflected an interplay between clinical information, personal values, and social influences. This theme focuses specifically on how patients understood W&W as a management option, weighed it against active treatment, and made or revisited decisions about their care. For some, W&W offered a rational, lower-risk alternative that preserved quality of life; for others, it felt counterintuitive or even like neglect. Trust in clinicians is considered here primarily in relation to decision-making: whether patients felt sufficiently informed, reassured and supported to accept monitoring rather than immediate treatment. Decisions were not fixed, and some patients later re-evaluated or abandoned W&W in light of anxiety, progression or changing circumstances. Four subthemes illustrate this process: choosing W&W over active treatment, influence of clinicians, family, peers and second opinions, counterintuitive perceptions of W&W, and regret, re-evaluation or switching to active treatment. Choosing W&W over active treatment.

Many described W&W as a deliberate weighing of risks and benefits. For those with low-grade disease or advanced age, it was seen as a logical, less disruptive alternative to surgery or chemotherapy. Active treatments were often perceived as more threatening than cancer itself, with fears of complications, long recovery, or diminished quality of life tipping the balance toward W&W. Some also valued its flexibility, allowing them to delay treatment without closing off future options. Yet even for those who embraced W&W, the choice was not straightforward; it required reconciling the comfort of avoiding treatment or “doing nothing” with the unease of living alongside cancer.

Influence of clinicians, family, peers and second opinions on decision-makingIn this subtheme, trust in clinicians was central because it shaped whether patients felt able to accept W&W as a credible and appropriate management strategy. Patients who received clear explanations and continuity of care often felt reassured, framing W&W as a shared decision. In contrast, rushed or dismissive interactions undermined confidence. Family and peers also shaped choices: partners sometimes urged active treatment out of concern, while friends’ or acquaintances’ experiences with cancer provided real-world comparisons that could sway decisions. Some patients sought second opinions to reduce uncertainty and make the “right” decision, underscoring uncertainty and the need for affirmation when making such a consequential choice.

### Counterintuitive perceptions of W&W

For some, W&W was initially perceived as contradictory to the very notion of cancer care. Patients described feeling bewildered or frustrated by the idea of having a diagnosis without active treatment, with several equating W&W with neglect or minimisation of their illness. This sense of contradiction was particularly acute when symptoms were absent, leading to disbelief that cancer even existed. Others reflected on how they struggled to accept W&W because it ran against cultural or personal expectations of cancer as something to be “fought” aggressively. Over time, however, some patients came to see W&W less as passive waiting and more as a proactive choice aligned with their values and health circumstances.

### Regret, re-evaluation, or switching to active treatment

Decisions around W&W often evolved. Some patients expressed regret, doubting whether they had chosen correctly, particularly when anxiety grew or when they observed others undergoing treatment. Others ultimately discontinued W&W, prompted by disease progression, clinical advice, or a personal desire to “take control” by removing cancer. For some, W&W was retrospectively viewed as merely delaying the inevitable; for others, it remained valuable, offering time free from treatment side effects. These varied experiences highlight the fluid nature of W&W decisions, which are continually re-evaluated in response to shifting medical, emotional, and social contexts.

### Theme 3: relationships and social experience

W&W was shaped not only by patients’ internal coping but also by their social worlds. The absence of symptoms or treatment often led to misunderstanding, minimisation, and feelings of invisibility. Some concealed their diagnosis to avoid stigma or dismissal, while others sought support from family, friends, or peers with cancer. These relational dynamics strongly influenced emotional wellbeing and strategies for managing uncertainty. Four subthemes capture these experiences: feeling misunderstood or isolated, minimisation by others, non-disclosure and concealment, and peer and family support.

### Feeling misunderstood or isolated

Patients frequently described a sense of being misunderstood, both by healthcare professionals and by people in their personal lives. Because they did not undergo active treatment, their struggles were often overlooked, leaving them feeling invisible or invalidated. Cultural expectations also played a role, with some describing pressures to “get on with it” or to hide vulnerability, which led to isolation. Living alone or lacking strong support networks heightened these challenges, leaving patients to cope privately with worry, uncertainty, and practical burdens. This social isolation was not only emotionally taxing but also reinforced feelings of being “different” from other cancer patients.

### Minimisation by others

The lack of symptoms and absence of treatment often led others (including clinicians, family, and friends) to minimize the seriousness of the diagnosis. Patients recalled being told they were “lucky” or that their cancer was “nothing to worry about,” which, while sometimes meant as reassurance, often felt dismissive and invalidating. Media portrayals of cancer as dramatic and life-threatening further contributed to the perception that W&W cancers were not “real cancers.” This minimisation was painful for many, as it denied the psychological toll of living with cancer and left them feeling excluded from the broader cancer community. For some, this lack of recognition became a source of frustration and resentment.

### Non-disclosure and social concealment

In the face of misunderstanding and minimisation, some patients chose not to disclose their diagnosis to others. Concealment was described as a way of protecting family members from worry, avoiding stigma, or unwanted pity. For those without symptoms, it was easier to keep cancer invisible, treating it as a private matter rather than a shared one. However, non-disclosure also carried emotional costs, often deepening feelings of loneliness and depriving patients of potential support. In this way, concealment could function as both a coping mechanism and a barrier to social connection.

### Peer and family support

Despite these challenges, many patients emphasized the importance of support from family, friends, and peers. Family support could be double-edged: sometimes providing reassurance and practical help, but at other times becoming overprotective or a source of pressure to pursue active treatment. Peer support, particularly through connecting with others undergoing W&W, was often described as uniquely valuable as it offered understanding, validation, and opportunities to share coping strategies. Encounters with peers, whether informally in waiting rooms or through organized groups, helped patients feel less alone and more equipped to manage the uncertainties of W&W. These findings highlight that, while relationships could exacerbate feelings of invisibility, they also provided critical resources for coping and adjustment.

## Theme 4: living with uncertainty

Uncertainty was a defining feature of living under W&W, shaping both everyday life and longer-term outlooks after the initial decision to monitor had been made. This theme focuses on how patients managed the continuing psychological burden of waiting, surveillance and not knowing. Patients described moving between reassurance and fear, attempting to balance normality with the awareness of cancer. In this theme, clinician communication and monitoring are considered in relation to ongoing uncertainty management rather than treatment decision-making. Monitoring could provide comfort through regular checks but also provoked anxiety, particularly when delayed or spaced far apart. Four subthemes illustrate these dynamics: lack of control and oscillation between calm and fear, living between illness and wellness, acute anxiety from monitoring procedures and results, and the psychological cycle of surveillance.

### Lack of control, oscillation between calm and fear

Patients frequently described uncertainty as a struggle between moments of reassurance and periods of fear. Here, clinician communication mattered because it shaped patients’ ability to tolerate ongoing uncertainty, rather than because it determined the initial decision to choose W&W. Some felt more able to manage uncertainty when clinicians communicated clearly, while others experienced frustration when information was limited or inconsistent. The sense of losing control over their health was prominent, with patients sometimes engaging in rituals or lifestyle adjustments in an attempt to regain agency. Yet these strategies often conflicted with the unpredictable nature of cancer, creating cycles of reassurance followed by renewed worry. This oscillation highlighted the fragile equilibrium patients tried to maintain between acceptance and anxiety.

### Living between illness and wellness

W&W placed patients in an in-between space, neither fully ill nor fully well. Many resumed daily routines, work, and social activities, emphasizing their ability to live a “normal” life despite the diagnosis. However, the presence of cancer disrupted this normality in subtle but persistent ways: fatigue, bodily awareness, or lifestyle restrictions (such as difficulties obtaining insurance) reminded them of their illness. For some, W&W reinforced the perception of cancer as a chronic, manageable condition, while for others, the invisibility of symptoms blurred the boundaries between health and illness. This ambiguous status created both opportunities for resilience and challenges to identity.

### Acute anxiety from monitoring procedures and results

Monitoring was described as both a source of reassurance and a trigger for anxiety. Anticipation of test results often evoked heightened fear, with some patients comparing monitoring appointments to “reliving the diagnosis.” While a clear or stable result could bring temporary relief, the reassurance was fragile, often lasting only until the next test was due. Delays or cancelations intensified distress, leaving patients feeling neglected or abandoned by the healthcare system. Others, however, described monitoring as routine, part of a process they accepted as necessary, or even reassuring because it provided structure and a sense of vigilance. Coping strategies varied, with some preferring less frequent testing to reduce anxiety, while others sought more frequent checks to maintain a sense of control.

### Psychological cycle of surveillance

The overarching challenge of W&W was the enduring weight of waiting. Patients described W&W as a prolonged state of limbo, characterized by uncertainty about disease progression, future treatments, and life expectancy. The lack of information (especially at the time of diagnosis) exacerbated this burden, leaving some feeling left in the dark and struggling to plan for the future. For others, the sense of carrying cancer “in the back of the mind” was unavoidable, shaping daily thoughts even when life outwardly appeared unchanged. This burden of waiting was sometimes experienced as more difficult than the cancer itself, underscoring the psychological costs of prolonged uncertainty.

## Theme 5: coping, control, and life adjustments

Patients adopted a range of strategies to cope with W&W and regain control in the face of uncertainty. These included lifestyle changes, reframing cancer to foster acceptance, seeking information or planning, and reprioritising life goals and identity. Coping approaches varied in effectiveness, revealing both resilience and vulnerability in how patients adapted over time.

### Lifestyle adjustments and health behaviors

Many patients responded to their diagnosis by adopting lifestyle changes they believed could positively influence their prognosis or give them a sense of control. These included changes in diet, exercise, stress management, or complementary therapies. While evidence for these strategies was sometimes uncertain, patients described them as empowering and a way of “doing something” in the face of watchful waiting. For some, these adjustments reduced anxiety and provided a sense of agency, whereas for others they became sources of guilt or pressure when goals were difficult to sustain.

### Reframing cancer and cultivating acceptance

Reframing cancer as indolent, chronic, or even as a condition that could be “lived with” was a central coping strategy. Patients described efforts to shift their mindset away from fear and toward acceptance, sometimes by emphasizing the benign nature of their disease or comparing themselves favorably to others with more aggressive cancers. Acceptance was often supported by clinician reassurance and time, as stability reinforced the idea that W&W was safe. However, reframing was not always stable; moments of fear or reminders of illness could unravel this sense of acceptance.

### Seeking agency through information or planning

Some patients coped by actively seeking information, researching treatment options, or planning for future contingencies. This forward planning provided comfort and reduced the sense of passivity associated with W&W. Others, however, found that too much information heightened anxiety, preferring instead to rely on clinicians’ expertise. Information-seeking was therefore both a tool for agency and a potential source of distress, highlighting the individual variability in how patients sought to regain control.

### Reprioritising life and identity

W&W often prompted patients to reassess priorities and reconfigure their sense of self. For some, the diagnosis served as a “wake-up call” that encouraged them to focus more on family, relationships, or meaningful activities. Others described a shift in identity, moving from viewing themselves primarily as patients to reclaiming roles as partners, workers, or community members. This reprioritisation was not always easy, particularly for those who continued to feel overshadowed by cancer, but for many it represented a key step in adaptation, enabling them to live more fully despite uncertainty.

## Discussion

### Findings in relation to previous research

This review synthesized qualitative evidence on the lived experience of patients undergoing W&W across cancer types. Five overarching themes captured the multifaceted psychosocial impact of W&W. Patients reported a spectrum of emotional responses, from initial shock and anxiety to identity struggles, acceptance, and avoidance. Decision-making was shaped by trust in clinicians, family and peer influence, and shifting perceptions of risk, with some revisiting choices over time. Social relationships were central: many experienced minimisation or misunderstanding, sometimes concealing their diagnosis, yet also drew strength from family and peer support. Uncertainty was a defining challenge, marked by oscillations between reassurance and fear, the liminal experience of being neither fully ill nor well, and the burden of monitoring and waiting. Finally, patients sought to regain control through coping strategies such as lifestyle changes, reframing cancer as manageable, and reprioritising life goals. Taken together, these findings highlight that W&W is not a passive state of “doing nothing,” but an active psychosocial process requiring continuous adaptation.

The contribution of this synthesis does not lie in claiming that each individual theme is wholly new to psycho-oncology. Rather, its contribution is in bringing together qualitative evidence across cancer types and W&W pathways to show that these experiences may form a broader cross-cancer psychosocial pattern. Anxiety, uncertainty, identity disruption, social misunderstanding, and reliance on clinician reassurance have each been described in specific cancer contexts, but this review demonstrates that they recur across different forms of monitored, untreated, or deferred-treatment cancer care. This suggests that W&W is not only a disease-specific clinical pathway, but also a shared psychosocial condition in which patients must live with cancer while being asked to wait, monitor, and adapt. This cross-cancer synthesis provides a basis for developing broader supportive-care principles, including clearer communication, validation of uncertainty, peer support, and psychological interventions that can be adapted across cancer pathways. Our findings reinforce and extend previous work demonstrating that W&W can be experienced as both a relief and a burden. Prior reviews have identified heightened risks of anxiety and depression among hematological patients managed under W&W compared to other cancer populations (Linden et al., 2012). Similarly, prostate cancer studies have highlighted unmet informational needs, the importance of trust in clinicians, and ambivalence around decision-making (Grassi et al., 2023). By synthesizing across diverse cancer types, this review uniquely demonstrates that these psychosocial challenges are not confined to a single diagnostic group but are fundamental to the W&W experience itself. This finding strongly suggests that cross-cancer supportive interventions are both feasible and necessary. Patients across conditions report oscillating between reassurance and fear, struggles with being seen as “real” cancer patients, the ongoing psychological weight of waiting, and the challenge of adapting life in the absence of active treatment.

In addition, this synthesis adds nuance by emphasizing coping and life adjustments as an integral part of the W&W experience. While previous studies have noted the role of acceptance and adaptation (Russell et al., 2022), these findings demonstrate the breadth of strategies patients employ ranging from lifestyle changes to meaning-making and reprioritisation of values. These adjustments align with findings in survivorship literature, where cancer prompts re-evaluation of identity and life goals (Habr-Gama et al., 2015). However, unlike traditional survivorship, patients on W&W remain under the shadow of active disease, complicating their efforts to construct stable narratives of health and identity.

### Strength and limitations

A strength of this review is its broad scope, integrating qualitative findings across cancer types to provide a comprehensive picture of the W&W experience. This allowed us to identify commonalities (such as anxiety about progression, struggles with identity, and lifestyle adaptation) that transcend diagnostic boundaries. The use of rigorous thematic synthesis methods also enabled the development of analytic themes that extend beyond the descriptive findings of individual studies.

However, several limitations should be acknowledged. Most included studies were conducted in high-income countries, limiting transferability to different cultural, socioeconomic and healthcare contexts. Although the broad themes identified in this review may be recognizable across settings, their form, intensity and practical consequences are likely to differ according to healthcare infrastructure, financial burden, family involvement, informal support, stigma and access to specialist oncology and psychosocial care. This is consistent with wider psycho-oncology literature showing that unmet psychosocial needs are shaped by access barriers and unequal availability of supportive care (Bergerot et al., 2024), and with evidence that cancer care can impose substantial financial toxicity in low- and middle-income countries (Donkor et al., 2022). Future research should therefore examine W&W experiences across more diverse geographical and socioeconomic contexts. Additionally, while synthesis allows for the identification of overarching patterns, nuances specific to individual cancer pathways may have been diluted.

### Clinical and research implications

These findings carry important implications for clinical practice. First, clinicians should be mindful that recommending W&W is not a neutral intervention but one that carries psychological consequences. Patients benefit when W&W is framed as an active, evidence-based choice rather than passive inaction. Clinician reassurance, continuity of care, and opportunities to ask questions repeatedly over time appear central to promoting acceptance and reducing anxiety.

Second, relational and social dimensions warrant greater attention. Structured peer support groups, either diagnosis-specific or cross-diagnostic, could provide validation and reduce feelings of invisibility. Incorporating family members into decision-making discussions may also mitigate tensions between patients’ preferences and relatives’ anxieties.

Third, psychological interventions tailored to W&W patients are needed. Our finding that patients often engage in denial or avoidance to manage distress provides a clear therapeutic target - interventions like Acceptance and Commitment Therapy (ACT) are designed to help patients by reducing unhelpful experiential avoidance and increasing psychological flexibility (the ability to draw on a broader and more workable repertoire of coping responses) (Arch et al., 2021; Van der Spek et al., 2017; Zhao et al., 2021; Brabbins et al., 2020). In addition, clinicians may wish to support patients’ efforts to regain control through lifestyle adjustments and future planning, recognizing these strategies as active coping rather than trivial lifestyle choices.

From a research perspective, future work should investigate longitudinal experiences of W&W, examining how psychological and social adjustment evolves over years rather than months. Greater attention is also required to diverse cancer types and underrepresented populations, as much of the existing literature is concentrated in prostate and hematological cancers. Additionally, evaluating psychosocial interventions specifically designed for W&W patients would provide evidence for targeted supportive care.

## Conclusion

This review demonstrates that W&W, while medically appropriate and often protective of quality of life, carries a distinctive psychosocial burden. Patients grapple with fluctuating anxiety, disrupted identity, relational misunderstandings, the enduring weight of uncertainty, and the challenge of reconstructing agency and meaning in daily life. Yet, many also adapt over time, reframing W&W as a manageable, even empowering, approach. To support this adaptation, clinicians and services must move beyond a biomedical framing of W&W to address its psychological and social dimensions. Tailored interventions, enhanced communication, and structured peer support represent promising avenues to improve the lived experience of patients on W&W pathways.
