# Supplementary material for: Living with Cancer under watch and wait: a qualitative systematic review and thematic synthesis
Source: Front Psychol. 2026 Oct 5;17:1830770. doi: 10.3389/fpsyg.2026.1830770 (PMC13677477; doi:10.3389/fpsyg.2026.1830770)
Supplement: Supplementary file 1 [file Supplementary_file_1.DOCX]

# Appendix 1: Search strategy and results from database searches

## 2.1 Search strategies

| **Platform and database**: Ovid MEDLINE® ALL <1946 to March 15, 2024> | | |
| --- | --- | --- |
| 1 | exp Neoplasms/ | 3947201 |
| 2 | (cancer* or tumor* or tumour* or neoplas* or carcinoma* or adenocarcinoma* or malignan* or oncolog* or sarcoma*).tw,kf. | 4265413 |
| 3 | (leukaemi* or leukemi* or lymphoma or myeloma or haematolog* or hematolog* or meningioma*).tw,kf. | 679551 |
| 4 | or/1-3 | 5432989 |
| 5 | Watchful Waiting/ | 5414 |
| 6 | ("watch and wait*" or "watch-and-wait*" or "watchful wait*" or "active surveillance" or "active monitor*" or "expectant management").tw,kf. | 19100 |
| 7 | or/5-6 | 21460 |
| 8 | 4 and 7 | 10530 |
| 9 | exp Psychological Distress/ | 7257 |
| 10 | (effect* or impact* or need* or distress or experience* or burden or "mental health").tw,kf. | 12325223 |
| 11 | or/9-10 | 12327389 |
| 12 | 8 and 11 | 4872 |
| 13 | exp Empirical Research/ or Interviews as Topic/ or Personal Narratives as Topic/ or Focus Groups/ or exp Narration/ or Nursing Methodology Research/ or Narrative Medicine/ | 185575 |
| 14 | (Interview or Personal Narrative).pt. | 37128 |
| 15 | (interview* or theme* or thematic or ethnological research or ethnograph* or ethnomedicine or ethnonursing or phenomenol* or life stor* or emic or etic or hermeneutic* or heuristic* or semiotic* or participant observ*).ti,ab,kf. | 624323 |
| 16 | qualitative.ti,ab,kf,jw. | 350438 |
| 17 | (grounded adj (theor* or study or studies or research or analys?s)).ti,ab,kf. | 16100 |
| 18 | (data adj1 saturat$).ti,ab,kf. | 2323 |
| 19 | (social construct* or postmodern* or post-structural* or post structural* or poststructural* or post modern* or post-modern* or action research or cooperative inquir* or co operative inquir* or co-operative inquir* or humanistic or existential or experiential or paradigm*).ti,ab,kf. | 213568 |
| 20 | (field adj (study or studies or research or work)).ti,ab,kf. | 22196 |
| 21 | (human science or social science or biographical method or theoretical sampl* or open-ended or narrative* or textual or texts or semi-structured or life world* or life-world* or conversation analys?s or personal experience* or theoretical saturation).ti,ab,kf. | 224814 |
| 22 | ((purpos* adj4 sampl*) or (focus adj group*)).ti,ab,kf. | 90633 |
| 23 | ((lived or life) adj experience*).ti,ab,kf. | 21335 |
| 24 | (cluster sampl* or observational method* or content analysis).ti,ab,kf. | 57670 |
| 25 | (constant adj (comparative or comparison)).ti,ab,kf. | 6279 |
| 26 | ((discourse* or discurs*) adj3 analys?s).ti,ab,kf. | 3454 |
| 27 | (heidegger* or colaizzi* or spiegelberg* or merleau* or husserl* or foucault* or ricoeur or glaser*).ti,ab,kf. | 5089 |
| 28 | (van adj manen*).ti,ab,kf. | 580 |
| 29 | (van adj kaam*).ti,ab,kf. | 45 |
| 30 | (corbin* adj2 strauss*).ti,ab,kf. | 471 |
| 31 | ((mixed or multiple) adj5 method*).ti,ab,kf. | 92366 |
| 32 | multimethod*.ti,ab,kf. | 2681 |
| 33 | or/13-32 | 1290495 |
| 34 | 12 and 33 | 343 |
| **Platform and database**: Ovide APA PsycInfo® <1806 to March Week 3 2024> | | |
| 1 | exp Neoplasms/ | 63579 |
| 2 | (cancer* or tumor* or tumour* or neoplas* or carcinoma* or adenocarcinoma* or malignan* or oncolog* or sarcoma*).tw,hw. | 101859 |
| 3 | (leukaemi* or leukemi* or lymphoma or myeloma or haematolog* or hematolog* or meningioma*).tw,hw. | 7267 |
| 4 | or/1-3 | 105557 |
| 5 | ("watch and wait*" or "watch-and-wait*" or "watchful wait*" or "active surveillance" or "active monitor*" or "expectant management").tw,hw. | 635 |
| 6 | 4 and 5 | 175 |
| 7 | Distress/ | 30937 |
| 8 | (effect* or impact* or need* or distress or experience* or burden or "mental health").tw,hw. | 2954397 |
| 9 | or/7-8 | 2954397 |
| 10 | 6 and 9 | 131 |
| 11 | Empirical Methods/ or exp Qualitative Methods/ or exp Interviews/ or Interviewing/ or Narratives/ or Storytelling/ | 75314 |
| 12 | (interview* or theme* or thematic or ethnological research or ethnograph* or ethnomedicine or ethnonursing or phenomenol* or life stor* or emic or etic or hermeneutic* or heuristic* or semiotic* or participant observ*).ti,ab,id. | 582812 |
| 13 | qualitative.ti,ab,jx,id. | 235482 |
| 14 | (grounded adj (theor* or study or studies or research or analys?s)).ti,ab,id. | 20802 |
| 15 | (data adj1 saturat$).ti,ab,id. | 595 |
| 16 | (social construct* or postmodern* or post-structural* or post structural* or poststructural* or post modern* or post-modern* or action research or cooperative inquir* or co operative inquir* or co-operative inquir* or humanistic or existential or experiential or paradigm*).ti,ab,id. | 185615 |
| 17 | (field adj (study or studies or research or work)).ti,ab,id. | 12900 |
| 18 | (human science or social science or biographical method or theoretical sampl* or open-ended or narrative* or textual or texts or semi-structured or life world* or life-world* or conversation analys?s or personal experience* or theoretical saturation).ti,ab,id. | 222066 |
| 19 | ((purpos* adj4 sampl*) or (focus adj group*)).ti,ab,id. | 61043 |
| 20 | ((lived or life) adj experience*).ti,ab,id. | 39042 |
| 21 | (cluster sampl* or observational method* or content analysis).ti,ab,id. | 37447 |
| 22 | (constant adj (comparative or comparison)).ti,ab,id. | 5878 |
| 23 | ((discourse* or discurs*) adj3 analys?s).ti,ab,id. | 11276 |
| 24 | (heidegger* or colaizzi* or spiegelberg* or merleau* or husserl* or foucault* or ricoeur or glaser*).ti,ab,id. | 9731 |
| 25 | (van adj manen*).ti,ab,id. | 669 |
| 26 | (van adj kaam*).ti,ab,id. | 560 |
| 27 | (corbin* adj2 strauss*).ti,ab,id. | 794 |
| 28 | ((mixed or multiple) adj5 method*).ti,ab,id. | 51147 |
| 29 | multimethod*.ti,ab,id. | 3957 |
| 30 | or/11-29 | 999283 |
| 31 | 10 and 30 | 42 |
| **Platform and database**: EBSCOhost Research Databases CINAHL Plus with Full Text | | |
| S1 | (MH "Neoplasms+") | 671,151 |
| S2 | TI (cancer* or tumor* or tumour* or neoplas* or carcinoma* or adenocarcinoma* or malignan* or oncolog* or sarcoma*) OR AB (cancer* or tumor* or tumour* or neoplas* or carcinoma* or adenocarcinoma* or malignan* or oncolog* or sarcoma*) | 752,256 |
| S3 | TI (leukaemi* or leukemi* or lymphoma or myeloma or haematolog* or hematolog* or meningioma*) OR AB (leukaemi* or leukemi* or lymphoma or myeloma or haematolog* or hematolog* or meningioma*) | 91,259 |
| S4 | S1 OR S2 OR S3 | 964,202 |
| S5 | TI ("watch and wait*" or "watch-and-wait*" or "watchful wait*" or "active surveillance" or "active monitor*" or "expectant management") OR AB ("watch and wait*" or "watch-and-wait*" or "watchful wait*" or "active surveillance" or "active monitor*" or "expectant management") | 5,524 |
| S6 | S4 AND S5 | 2,350 |
| S7 | (MH "Psychological Distress") | 7,012 |
| S8 | TI (effect* or impact* or need* or distress or experience* or burden or "mental health") OR AB (effect* or impact* or need* or distress or experience* or burden or "mental health") | 2,570,338 |
| S9 | S7 OR S8 | 2,571,249 |
| S10 | S6 AND S9 | 1,067 |
| S11 | MH Qualitative Studies OR MH Grounded theory OR MH Narratives OR MH Interviews+ OR MH Audiorecording OR MH Focus Groups OR MH Research, Nursing OR MH Discourse Analysis OR MH Content Analysis OR MH Ethnographic Research OR MH Ethnonursing Research OR MH Constant Comparative Method OR MH Qualitative Validity+ OR MH Purposive Sample OR MH Observational Methods+ OR MH Field Studies OR MH Theoretical Sample OR MH Phenomenology OR MH Phenomenological Research OR MH Life Experiences+ OR MH Cluster Sample+ | 463,592 |
| S12 | TI (interview* or theme* or thematic or "ethnological research" or ethnograph* or ethnomedicine or ethnonursing or phenomenol* or "life stor*" or emic or etic or hermeneutic* or heuristic* or semiotic* or "participant observ*" or qualitative* or "focus group*") OR AB (interview* or theme* or thematic or "ethnological research" or ethnograph* or ethnomedicine or ethnonursing or phenomenol* or "life stor*" or emic or etic or hermeneutic* or heuristic* or semiotic* or "participant observ*" or qualitative* or "focus group*") | 417,796 |
| S13 | TI (grounded N1 (theor* OR analys?s OR research OR studies OR study)) OR AB (grounded N1 (theor* OR analys?s OR research OR studies OR study)) | 15,224 |
| S14 | TI (data N1 saturat*) OR AB (data N1 saturat*) | 1,474 |
| S15 | TI (social construct* or postmodern* or post-structural* or "post structural*" or poststructural* or "post modern*" or post-modern* or "action research" or "cooperative inquir*" or "co operative inquir*" or "co-operative inquir*" or humanistic or existential or experiential or paradigm*) OR AB (social construct* or postmodern* or post-structural* or "post structural*" or poststructural* or "post modern*" or post-modern* or "action research" or "cooperative inquir*" or "co operative inquir*" or "co-operative inquir*" or humanistic or existential or experiential or paradigm*) | 56,511 |
| S16 | TI (field N1 (research OR study OR studies)) OR AB (field N1 (research OR study OR studies)) | 7,695 |
| S17 | TI ("human science" or "social science" or "biographical method" or "theoretical sampl*" or open-ended or narrative* or textual or texts or semi-structured or "life world*" or life-world* or "conversation analys?s" or "personal experience*" or "theoretical saturation") OR AB ("human science" or "social science" or "biographical method" or "theoretical sampl*" or open-ended or narrative* or textual or texts or semi-structured or "life world*" or life-world* or "conversation analys?s" or "personal experience*" or "theoretical saturation") | 160,378 |
| S18 | TI (purpos* N4 sampl*) OR AB (purpos* N4 sampl*) | 16,698 |
| S19 | TI ((life OR lived) N1 experience*) OR AB ((life OR lived) N1 experience*) | 19,224 |
| S20 | TI ("cluster sampl*" or "observational method*" or "content analysis") OR AB ("cluster sampl*" or "observational method*" or "content analysis") | 37,008 |
| S21 | TI ((discurs* OR discourse*) N3 analys?s) OR AB ((discurs* OR discourse*) N3 analys?s) | 3,292 |
| S22 | TI (constant N1 (comparison OR comparative)) OR AB (constant N1 (comparison OR comparative)) | 5,458 |
| S23 | TI (heidegger* or colaizzi* or spiegelberg* or merleau* or husserl* or foucault* or ricoeur or glaser*) OR AB (heidegger* or colaizzi* or spiegelberg* or merleau* or husserl* or foucault* or ricoeur or glaser*) | 4,731 |
| S24 | TI (van N1 manen*) OR AB (van N1 manen*) | 849 |
| S25 | TI (van N1 kaam*) OR AB (van N1 kaam*) | 75 |
| S26 | TI (Corbin* N2 strauss*) OR AB (Corbin* N2 strauss*) | 481 |
| S27 | TI ((mixed or multiple) N5 method*) OR AB (mixed or multiple) N5 method*) | 41,262 |
| S28 | TI (multimethod*) OR AB (multimethod*) | 1,177 |
| S29 | S11 OR S12 OR S13 OR S14 OR S15 OR S16 OR S17 OR S18 OR S19 OR S20 OR S21 OR S22 OR S23 OR S24 OR S25 OR S26 OR S27 OR S28 | 767,157 |
| S30 | S10 AND S29 | 140 |

## 2.2 Results of the database searches

| **Database** | **Platform** | **Results before duplicates removed** | **Results after duplicates removed** | **Date of search** |
| --- | --- | --- | --- | --- |
| MEDLINE® ALL | Ovid <1946 to January 03, 2024> | 343 | 316 | 18 Mar 2024 |
| APA PsycInfo® |  | 42 | 14 | 18 Mar 2024 |
| CINAHL Plus with Full Text | EBSCOhost <1987 to present> | 140 | 63 | 18 Mar 2024 |
| Total number of results retrieved | | 525 |  |  |
| Number of duplicates removed | | 132 |  |  |
| **Total number of results to screen** | |  | 393 |  |

# Appendix 2: Quality Appraisal

|  | CASP | Al Hussein et al., 2023 | Bailey Jr et al., 2007 | Beckmann et al., 2021 | Berger et al., 2014 | Charbonneau et al., 2022 | Deobald et al., 2015 | Donachie et al., 2020 | Dunnion et al., 2023 | Evans et al., 2011 | Eymech et al., 2022 | Hermus et al., 2022 | Kyrou et al., 2024 | Mader et al., 2017 | Matheson et al., 2019 | McCaughan et al., 2023 | McIntosh et al., 2022 | Pennings et al., 2021 | Russell et al., 2023 | Seaman et al., 2019 | Tan et al., 2014 | Wade et al., 2020 | Wiener et al., 2012 | Zanatto et al., 2023 |
| --- | --- | --- | --- | --- | --- | --- | --- | --- | --- | --- | --- | --- | --- | --- | --- | --- | --- | --- | --- | --- | --- | --- | --- | --- |
| 1 | Was there a clear statement of the aims of the research? | Yes | Yes | Yes | Yes | Yes | No | Yes | Yes | Yes | Yes | Yes | Yes | Yes | Yes | Yes | Yes | Yes | Yes | Yes | Yes | Yes | Yes | Yes |
| 2 | Is a qualitative methodology appropriate? | Yes | Yes | Yes | Yes | Yes | Yes | Yes | Yes | Yes | Yes | Yes | Yes | Yes | Yes | Yes | Yes | Yes | Yes | Yes | Yes | Yes | Yes | Yes |
| 3 | Was the research design appropriate to address the aims of the research? | Yes | Yes | Yes | Yes | Yes | Yes | Yes | Yes | Yes | Yes | Yes | Yes | Yes | Yes | Yes | Yes | Yes | Yes | Yes | Yes | Yes | Yes | Yes |
| 4 | Was the recruitment strategy appropriate to the aims of the research? | Yes | Yes | Yes | Yes | Yes | Yes | Yes | Yes | Yes | Yes | Yes | Yes | Yes | Yes | Yes | Yes | Yes | Yes | Yes | Yes | Yes | Yes | Yes |
| 5 | Was the data collected in a way that addressed the research issue? | Yes | Yes | Yes | Yes | Yes | Yes | Yes | Yes | Yes | Yes | Yes | Yes | Yes | Yes | Yes | Yes | Yes | Yes | Yes | Yes | Yes | Yes | Yes |
| 6 | Has the relationship between researcher and participants been adequately considered? | No | No | No | No | No | Yes | No | No | No | Yes | No | Yes | No | No | No | No | Yes | No | Yes | No | No | No | No |
| 7 | Have ethical issues been taken into consideration? | Yes | ? | Yes | Yes | Yes | Yes | Yes | Yes | Yes | Yes | Yes | Yes | Yes | Yes | Yes | Yes | Yes | Yes | Yes | Yes | Yes | Yes | Yes |
| 8 | Was the data analysis sufficiently rigorous? | Yes | Yes | Yes | Yes | Yes | Yes | Yes | Yes | Yes | Yes | Yes | Yes | Yes | Yes | Yes | Yes | Yes | Yes | Yes | Yes | Yes | Yes | Yes |
| 9 | Is there a clear statement of findings? | Yes | Yes | Yes | Yes | Yes | Yes | Yes | Yes | Yes | Yes | Yes | Yes | Yes | Yes | Yes | Yes | Yes | Yes | Yes | Yes | Yes | Yes | Yes |
| 1 0 | How valuable is the research? | Yes | Yes | No | Yes | Yes | Yes | Yes | Yes | Yes | Yes | No | Yes | Yes | Yes | Yes | Yes | Yes | Yes | Yes | Yes | Yes | Yes | Yes |
|  |  |  |  |  |  |  |  |  |  |  |  |  |  |  |  |  |  |  |  |  |  |  |  |  |
|  |  |  |  |  |  |  |  |  |  |  |  |  |  |  |  |  |  |  |  |  |  |  |  |  |
|  |  |  |  |  |  |  |  |  |  |  |  |  |  |  |  |  |  |  |  |  |  |  |  |  |
|  |  |  |  |  |  |  |  |  |  |  |  |  |  |  |  |  |  |  |  |  |  |  |  |  |
|  |  |  |  |  |  |  |  |  |  |  |  |  |  |  |  |  |  |  |  |  |  |  |  |  |
|  |  |  |  |  |  |  |  |  |  |  |  |  |  |  |  |  |  |  |  |  |  |  |  |  |
|  |  |  |  |  |  |  |  |  |  |  |  |  |  |  |  |  |  |  |  |  |  |  |  |  |
|  |  |  |  |  |  |  |  |  |  |  |  |  |  |  |  |  |  |  |  |  |  |  |  |  |
|  |  |  |  |  |  |  |  |  |  |  |  |  |  |  |  |  |  |  |  |  |  |  |  |  |
|  |  |  |  |  |  |  |  |  |  |  |  |  |  |  |  |  |  |  |  |  |  |  |  |  |
|  |  |  |  |  |  |  |  |  |  |  |  |  |  |  |  |  |  |  |  |  |  |  |  |  |
|  |  |  |  |  |  |  |  |  |  |  |  |  |  |  |  |  |  |  |  |  |  |  |  |  |
|  |  |  |  |  |  |  |  |  |  |  |  |  |  |  |  |  |  |  |  |  |  |  |  |  |
